# Leveraging Multimedia Patient Engagement to Address Minority Cerebrovascular Health Needs: Prospective Observational Study

**DOI:** 10.2196/28748

**Published:** 2021-08-13

**Authors:** Elizabeth Anne Noser, Jing Zhang, Mohammad Hossein Rahbar, Anjail Zarinah Sharrief, Andrew David Barreto, Sandi Shaw, James Charles Grotta, Sean Isaac Savitz, Nneka Lotea Ifejika

**Affiliations:** 1 Institute for Stroke and Cerebrovascular Disease UTHealth Houston, TX United States; 2 Department of Neurology McGovern Medical School at UTHealth Houston, TX United States; 3 Department of Internal Medicine Division of Clinical & Translational Sciences McGovern Medical School at UTHealth Houston, TX United States; 4 Department of Biostatistics and Data Science UTHealth School of Public Health Houston, TX United States; 5 Mischer Neuroscience Institute Memorial Hermann Hospital - Texas Medical Center Houston, TX United States; 6 Stroke Research and Mobile Stroke Unit Memorial Hermann Hospital - Texas Medical Center Houston, TX United States; 7 Department of Physical Medicine and Rehabilitation UT Southwestern Medical Center Dallas, TX United States; 8 Department of Neurology UT Southwestern Medical Center Dallas, TX United States; 9 Department of Population and Data Sciences UT Southwestern Medical Center Dallas, TX United States

**Keywords:** environmental justice, urban flooding, stroke, community engagement, education, health disparities

## Abstract

**Background:**

Social inequities affecting minority populations after Hurricane Katrina led to an expansion of environmental justice literature. In August 2017, Hurricane Harvey rainfall was estimated as a 3000- to 20,000-year flood event, further affecting minority populations with disproportionate stroke prevalence. The Stomp Out Stroke initiative leveraged multimedia engagement, creating a patient-centered cerebrovascular health intervention.

**Objective:**

This study aims to address social inequities in cerebrovascular health through the identification of race- or ethnicity-specific health needs and the provision of in-person stroke prevention screening during two community events (May 2018 and May 2019).

**Methods:**

Stomp Out Stroke recruitment took place through internet-based channels (websites and social networking). Exclusively through web registration, Stomp Out Stroke participants (aged >18 years) detailed sociodemographic characteristics, family history of stroke, and stroke survivorship. Participant health interests were compared by race or ethnicity using Kruskal-Wallis or chi-square test at an α=.05. A Bonferroni-corrected *P* value of .0083 was used for multiple comparisons.

**Results:**

Stomp Out Stroke registrants (N=1401) were 70% (973/1390) female (median age 45 years) and largely self-identified as members of minority groups: 32.05% (449/1401) Hispanic, 25.62% (359/1401) African American, 13.63% (191/1401) Asian compared with 23.63% (331/1401) non-Hispanic White. Stroke survivors comprised 11.55% (155/1401) of our population. A total of 124 stroke caregivers participated. Approximately 36.81% (493/1339) of participants had a family history of stroke. African American participants were most likely to have Medicare or Medicaid insurance (84/341, 24.6%), whereas Hispanic participants were most likely to be uninsured (127/435, 29.2%). Hispanic participants were more likely than non-Hispanic White participants to obtain *health screenings* (282/449, 62.8% vs 175/331, 52.9%; *P*=.03). Asian (105/191, 54.9%) and African American (201/359, 55.9%) participants were more likely to request *stroke education* than non-Hispanic White (138/331, 41.6%) or Hispanic participants (193/449, 42.9%). African American participants were more likely to seek *overall health education* than non-Hispanic White participants (166/359, 46.2% vs 108/331, 32.6%; *P*=.002). Non-Hispanic White participants (48/331, 14.5%) *were less likely to speak to health care providers* than African American (91/359, 25.3%) or Asian participants (54/191, 28.3%). During the 2018 and 2019 events, 2774 health screenings were completed across 12 hours, averaging four health screenings per minute. These included blood pressure (1031/2774, 37.16%), stroke risk assessment (496/2774, 17.88%), bone density (426/2774, 15.35%), carotid ultrasound (380/2774, 13.69%), BMI (182/2774, 6.56%), serum lipids (157/2774, 5.65%), and hemoglobin A_1c_ (102/2774, 3.67%). Twenty multimedia placements using the Stomp Out Stroke webpage, social media, *#stompoutstroke*, television, iQ radio, and web-based news reached approximately 849,731 people in the Houston area.

**Conclusions:**

Using a combination of internet-based recruitment, registration, and in-person assessments, Stomp Out Stroke identified race- or ethnicity-specific health care needs and provided appropriate screenings to minority populations at increased risk of urban flooding and stroke. This protocol can be replicated in Southern US *Stroke Belt* cities with similar flood risks.

## Introduction

### Importance of the Problem

Social inequities affecting minority populations after Hurricane Katrina led to an expansion of the environmental justice literature after large-scale floods [1]. In 2017, Hurricane Harvey rainfall over a 4-day period was estimated as a 3000- and 20,000-year flooding event [2]. Studies after Hurricane Harvey in Houston detailed more extensive flooding among racial or ethnic minority households and those of lower socioeconomic status [3,4].

Houston is not only ranked among the most flood-impacted urban centers in the United States but also has some of the highest national stroke mortality rates, significantly affecting minority health [5]. In Harris County, Texas, which encompasses Houston, 4.7% of Medicare beneficiaries have been treated for stroke, placing Harris County in the worst quartile of all counties in the United States [6]. The combined effects of flood impact and cerebrovascular disease disparities are a growing concern for environmental justice. Correlated with the underdiagnosis and undertreatment of risk factors, minorities have a median age of first stroke 10-13 years earlier than non-Hispanic White people [5,7]. The age-adjusted incidence of first ischemic stroke is 179 per 100,000 in non-Hispanic White people, compared with 294 per 100,000 in African American people [8]. Direct medical costs for African American patients with stroke were estimated to reach 16 billion dollars in 2020; by 2030, stroke prevalence is expected to rise the most among Hispanic men, with direct costs of care increasing over 300%, compared with 140% in non-Hispanic White men, since 2012 [9].

### Pros and Cons of Multimedia Stroke Prevention in Minority Populations

Minority populations are receptive to the use of mobile health (mHealth) technology for health interventions; however, racial differences in technology use and internet access persist [10]. In a recent study of stroke survivors and caregivers by Naqvi et al [11], the highest number of participants with reported internet access were non-Hispanic White people. Furthermore, studies on stroke risk factor management using mobile apps versus usual care in minority populations have yielded equivocal results [12].

### Purpose

Studies have shown that ongoing public education on stroke symptoms improves stroke recognition [13,14]; unfortunately, racial disparities continue to exist regarding stroke literacy [15,16].

There are limited data regarding the practice of community engagement in stroke systems of care in flood-prone areas [17], including the method of delivery and educational activities. Furthermore, the utilization of a hybrid multimedia model—using technology to identify health care needs specific to minority populations, followed by in-person health screenings—has not been evaluated.

The purpose of this study was to implement Stomp Out Stroke, a hybrid multimedia education and health screening paradigm, serving a population disproportionately affected by Hurricane Harvey flooding. We hypothesize that the Stomp Out Stroke structure identifies and provides targeted health interventions, fulfilling specific needs, stratified by race and ethnicity. Multiple coastal cities in the Southern *Stroke Belt* (New Orleans, Louisiana; Charleston, South Carolina; and Savannah, Georgia) have similar demographics and flood risk, improving the generalizability of this population health intervention.

## Methods

### Conceptualization

**Figure 1:**
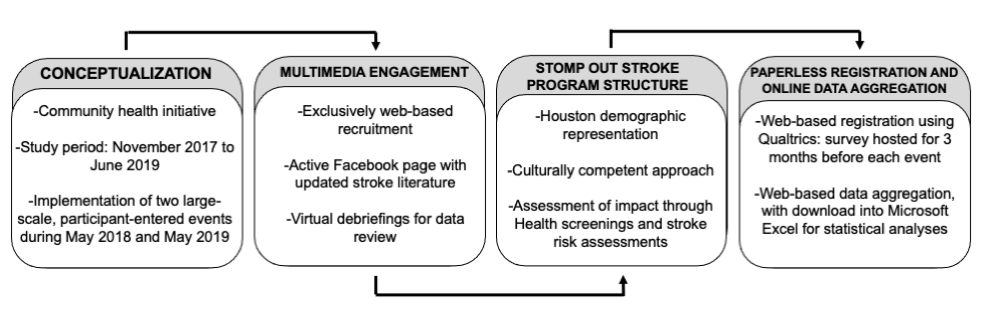
Stomp Out Stroke flow process.

Stomp Out Stroke is a prospective observational study and a collaborative public education initiative, which was implemented by the Vascular Neurology Program at the McGovern Medical School at the University of Texas Health Science Center at Houston (UTHealth) as part of the Joint Commission–Certified Integrated Stroke Healthcare System at Memorial Hermann Health System. Both institutions are located in the 2.1 square mile Texas Medical Center, topographically distributed within the 100-year and 500-year floodplains due to Brays Bayou, a slow-moving river that borders the health care district [18]. Stomp Out Stroke study period was from November 2017 to June 2019, including patient and public involvement, program structure, and implementation of two events: the first in May 2018 and the second in May 2019. Figure 1 details the Stomp Out Stroke flow process.

### Multimedia Engagement

Recruitment for participation in Stomp Out Stroke occurred exclusively via web-based platforms. Both the Texas Medical Center and the Texas Heart Institute provided location information, types of activities, and links to the registration website [19-21]. Stomp Out Stroke also has an active Facebook page, which is consistently updated with stroke awareness literature [22]. We used the social media hashtag *#stompoutstroke* on Twitter, Instagram, and Facebook. Members of the Vascular Neurology program also participated in television, iQ radio, and web-based news interviews to expand our reach.

Evidence-based protocols for primary stroke prevention were followed using the US Preventive Services Task Force Guide to Clinical Preventive Services [23] and the American Heart Association Guidelines for Heart-Health Screenings [24]. The Alzheimer’s Foundation of America National Memory Screening program detailed the protocols for cognitive testing [25]. Virtual Stomp Out Stroke debriefings facilitated the review of data on attendee health screenings, volunteer or exhibitor evaluations, and opportunities for growth.

### Stomp Out Stroke Program Structure

#### Houston Demographics

Houston is the fourth largest city in the United States, with a 2018 population estimate of 2.3 million people [26]. The Stomp Out Stroke program structure reflected Houston’s racial or ethnic diversity and socioeconomic characteristics. The approximate racial or ethnic makeup of Houston is 45% Hispanic or Latino, 22.09% African American, 24.41% non-Hispanic White, and 6.72% Asian [27]. Overall, 30% of Houston residents were born outside of the United States. Twenty-five percent of people aged <65 years are uninsured and 20% live in poverty.

#### Content

Stomp Out Stroke was divided into five zones: central, stage and entertainment, healthy brain, stroke recovery, and children’s. Each zone had health education and screening stations and was staffed by a volunteer coordinator and co-coordinator. Family-friendly activities and entertainment, including local multicultural dance groups, were included throughout the program (Multimedia Appendix 1).

#### Culturally Competent Approach

To address disparities in stroke literacy due to language barriers, bilingual health care providers and students were recruited as volunteers to assist in registration or check-in and to conduct health screenings or risk assessments in Spanish, simplified Chinese, and Vietnamese.

#### Assessment of Impact: Health Screenings or Stroke Risk Assessments

Assessment of impact was determined through the completion of onsite health screenings and stroke risk assessments. Each health screening consisted of vascular risk factor counseling, risk modification strategies, and recommended primary care provider follow-up. At the end of each screening, participants received printed educational materials and a give-away item (value <US $5). Automated blood pressure monitors (HEM-7222-ITZ; Omron Healthcare, Inc) were used to obtain a single measure. Additional health screenings included bone density, carotid ultrasound, BMI, serum lipids, and hemoglobin A_1c._ The 10-year stroke risk was calculated utilizing a modified Framingham stroke risk profile assessment tool, which contains age, sex, and baseline measurements of cerebrovascular risk factors, including systolic blood pressure, use of antihypertensive medications, current smoking status, presence of cardiovascular disease, current or prior atrial fibrillation, and diabetes mellitus (Multimedia Appendix 2) [28,29].

### Paperless Registration and Web-Based Data Aggregation

Institutional review board approval was obtained at the McGovern Medical School at UTHealth to collect voluntary onsite and web-based registration data (HSC-MS-15-0813—UT Stroke Team Community Outreach Program). The UTHealth Institute for Stroke and Cerebrovascular Diseases has an archive of past Stomp Out Stroke events, detailing the evolution of the program before the study period [30]. During the 3 months before the event, the *Register Here* button on the front page linked participants to a web-based registration form using Qualtrics software, Version March 2020 [31]. No paper registration forms were used. Registration forms (aged >18 years) included sociodemographics, stroke survivorship, caregiver of a stroke survivor, or family history of stroke. Participants’ interest in attending Stomp Out Stroke was assessed. On the event day, two check-in stations were available for preregistrants, and two stations were available for onsite registration through Qualtrics. At the end of both the 2018 and 2019 events, Qualtrics surveys were closed, and participant responses were downloaded into Microsoft Excel spreadsheets for analyses.

### Statistical Analysis

Sociodemographic characteristics were summarized using frequency and percentage or median and IQR for nonnormal distributions. Comparisons by racial group or ethnicity (Asian, Black or African American, Hispanic, or non-Hispanic White) were conducted using the Kruskal-Wallis (for age) or chi-square test. A *P* value of <.05 indicated a statistically significant difference between at least two of the four ethnic groups. A Bonferroni-corrected *P* value of .0083 (.05 divided by 6) was used to control for multiple comparisons. If the pairwise comparison *P* value was <.0083, there was a statistically significant difference between multiple ethnic groups. All analyses were performed using SAS (version 9.4; SAS Institute Inc).

## Results

### Overview

Table 1 details the sociodemographic characteristics of Stomp Out Stroke registrants. Data from 1401 Stomp Out Stroke registrants in 2018 and 2019 were analyzed. Less than 5% of the data were missing, and imputation was not used.

**Table 1:** Summary of sociodemographic characteristics: 2018 and 2019 Stomp Out Stroke registrants (N=1401).

Variable		Value
Age in years (n=1381), median (IQR)		45.0 (36-57)
**Sex (n=1390), n (%)**		
	Female	973 (70)
	Male	417 (30)
**Marital status (n=1383), n (%)**		
	Married	770 (55.68)
	Not married	613 (44.32)
**Race or ethnicity (n=1401), n (%)**		
	Hispanic	449 (32.05)
	Black or African American	359 (25.62)
	Non-Hispanic White	331 (23.63)
	Asian	191 (13.63)
	Other, two, or more races	49 (3.5)
	American Indian, Alaska Native, Native American, or Pacific Islander	5 (0.36)
	Unknown	17 (1.21)
**Hispanic ethnicity (n=449), n (%)**		
	White	234 (52.12)
	Black or African American	9 (2)
	American Indian, Alaska Native, Native American, or Pacific Islander	8 (1.78)
	Asian	3 (0.67)
	Other, two, or more races	136 (30.3)
	Unknown	59 (13.29)
**Have children (n=1389), n (%)**		
	No	882 (63.5)
	Yes	507 (36.5)
**Health insurance (n=1342), n (%)**		
	Employer based	614 (45.75)
	Medicare or Medicaid	251 (18.7)
	Private insurance	205 (15.28)
	Self-insured	44 (3.28)
	Uninsured	228 (16.99)
**Education (n=1331), n (%)**		
	More than high school	1053 (79.11)
	High school or less	278 (20.89)
**Are you a stroke survivor? (n=1342), n (%)**		
	No	1187 (88.45)
	Yes	155 (11.55)
**Are you a caregiver for a stroke survivor? (n=1339), n (%)**		
	No	1215 (90.74)
	Yes	124 (9.26)
**Do you have a family member who is a stroke survivor? (n=1339), n (%)**		
	No	846 (63.18)
	Yes	493 (36.81)

The median registrant age was 45 years; 69.54% (973/1390) were female and 55.68% (770/1383) were married. Overall, 32.05% (449/1401) of registrants self-identified as Hispanic, 25.62% (359/1401) self-identified as Black or African American, 23.63% (331/1401) self-identified as a non-Hispanic White person, and 13.63% (191/1401) self-identified as Asian.

A total of 63.5% (882/1389) of Stomp Out Stroke participants did not have children, 79.11% (1053/1331) had more than a high school education, and 45.75% (614/1342) had employer-based health insurance. Moreover, 155 Stomp Out Stroke participants were self-reported stroke survivors and 124 were caregivers. Nearly 36.81% (493/1339) of the registrants had a family history of stroke (Table 1).

Table 2 compares the sociodemographic characteristics of Stomp Out Stroke participants by racial or ethnic group. Hispanic participants were significantly younger than African American or non-Hispanic White participants (aged 43 years vs 48 or 49 years). A larger proportion of African American women participants were noted in comparison with Asian or non-Hispanic White people (274/358, 76.5% vs 124/188, 66% or 216/330, 65.5%).

Hispanic participants were the least likely among the four ethnic groups to have education past high school (274/432, 63.4% vs 269/322, 83.5% non-Hispanic White; 291/338, 86.1% African American; and 164/176, 93.2% Asian participants), and African American participants were the least likely to be married (128/357, 35.9% vs 200/329, 60.8% non-Hispanic White; 274/443, 61.9% Hispanic; and 123/189, 65.1% Asian participants; Table 2).

African American participants were the most likely to have Medicare or Medicaid insurance 24.6% (84/341), whereas Hispanic participants were the most likely to be uninsured 29.2% (127/435). In total, 19.9% (68/341) of African American registrants were stroke survivors; this group was the most likely to have a family history of stroke 46.5% (158/340) and more likely than non-Hispanic White or Hispanic people to have previously participated in Stomp Out Stroke (Table 2).

**Table 2:** Sociodemographic characteristics of Stomp Out Stroke participants by racial or ethnic group (N=1401).

Variable		Asian	Black or African American	Non-Hispanic White	Hispanic	*P* value^a^	
**Age (years)**							<.001
	Participants, n	187	353^b^	330^c^	443^b,c^		
	Value, median (IQR)	45.0 (34.0-55.0)	48.0 (38.0-58.0)	49.0 (36.0-60.0)	43.0 (34.0-55.0)		
**Sex, n (%)**							.01
	Participants	188 (100)^b^	358 (100)^b,c^	330 (100)^c^	446 (100)		
	Female	124 (66)	274 (76.5)	216 (65.5)	311 (69.7)		
	Male	64 (34)	84 (23.5)	114 (34.5)	135 (30.3)		
**Education, n (%)**							<.001
	Participants	176 (100)^b,c^	338 (100)^d^	322 (100)^b,e^	432 (100)^c,d,e^		
	More than high school	164 (93.2)	291 (86.1)	269 (83.5)	274 (63.4)		
	High school or less	12 (6.8)	47 (13.9)	53 (16.5)	158 (36.6)		
**Marital status, n (%)**							<.001
	Participants	189 (100)^b^	357 (100)^b,c,d^	329 (100)^c^	443 (100)^d^		
	Married	123 (65.1)	128 (35.9)	200 (60.8)	274 (61.9)		
	Not married	66 (34.9)	229 (64.1)	129 (39.2)	169 (38.1)		
**Health insurance, n (%)**							<.001
	Participants	180 (100)^b,c^	341 (100)^b,d,e^	322 (100)^d,f^	435 (100)^c,e,f^		
	Employer based	107 (59.4)	151 (44.3)	182 (56.5)	144 (33.1)		
	Medicare or Medicaid	16 (8.9)	84 (24.6)	53 (16.5)	87 (20)		
	Private insurance	22 (12.2)	50 (14.7)	49 (15.2)	70 (16.1)		
	Self-insured	11 (6.1)	11 (3.2)	13 (4)	7 (1.6)		
	Uninsured	24 (13.3)	45 (13.2)	25 (7.8)	127 (29.2)		
**Have children, n (%)**							.05
	Participants	191 (100)	355 (100)	329 (100)	448 (100)		
	No	135 (70.7)	225 (63.4)	220 (66.9)	270 (60.3)		
	Yes	56 (29.3)	130 (36.6)	109 (33.1)	178 (39.7)		
**Are you a stroke survivor? n (%)**							<.001
	Participants	180 (100)^b,c^	341 (100)^b,d,e^	321 (100)^c,d^	438 (100)^e^		
	No	173 (96.1)	273 (80.1)	284 (88.5)	404 (92.2)		
	Yes	7 (3.9)	68 (19.9)	37 (11.5)	34 (7.8)		
**Are you a caregiver for a stroke survivor? n (%)**							.03
	Participants	178 (100)^b^	339 (100)	323 (100)^b^	435 (100)		
	No	152 (85.4)	304 (89.7)	301 (93.2)	399 (91.7)		
	Yes	26 (14.6)	35 (10.3)	22 (6.8)	36 (8.3)		
**Do you have a family member who is a stroke survivor? n (%)**							<.001
	Participants	178 (100)^b^	340 (100)^b,c,d^	322 (100)^c^	435 (100)^d^		
	No	117 (65.7)	182 (53.5)	211 (65.5)	292 (67.1)		
	Yes	61 (34.3)	158 (46.5)	111 (34.5)	143 (32.9)		
**Previously attended stroke festival? n (%)**							<.001
	Participants	181 (100)	343 (100)^b,c^	323 (100)^b^	439 (100)^c^		
	No	136 (75.1)	221 (64.4)	256 (79.3)	353 (80.4)		
	Yes	45 (24.9)	122 (35.6)	67 (20.7)	86 (19.6)		

^a^Except for the *P* value in the Age row, which is based on the Kruskal-Wallis test, all remaining *P* values are based on the chi-square test.

^b-f^Indicate significant differences in pairwise comparisons of either median scores or proportions in different ethnic groups. The presence of the same letter in the columns for two race or ethnic groups (ie, “a” in the Asian and “a” in the African-American race columns) indicate a significant difference between the median scores or proportions between those two respective ethnic groups.

Table 3 compares interest in attending Stomp Out Stroke by racial or ethnic group. African American registrants were more likely to attend Stomp Out Stroke for family fun and entertainment than Asian registrants (173/359, 48.2% vs 66/191, 34.6%). Hispanic registrants were more likely than non-Hispanic White people to attend Stomp Out Stroke for free health screenings (282/449, 62.8% vs 175/331, 52.9%). Asian and African American registrants were more likely to attend Stomp Out Stroke for stroke education than non-Hispanic White or Hispanic people (105/191, 54.9% Asian or 201/359, 55.9% African American vs 138/331, 41.7% non-Hispanic White or 193/449, 42.9% Hispanic). African American participants were more likely to express interest in learning about health care topics other than stroke than non-Hispanic White people (166/359, 46.2% vs 108/331, 32.6%). Non-Hispanic White people were less likely than African American or Asian registrants to speak to a health care provider (48/331, 14.5% non-Hispanic White vs 91/359, 25.3% African American or 54/191, 28.3% Asian).

**Table 3:** Participant interest in attending Stomp Out Stroke by racial or ethnic group (n=1330).

Variable			Asian		Black or African American		Non-Hispanic White		Hispanic		*P* value^a^	
**Family fun and entertainment, n (%)**												.01
	Participants	191 (100)^b^		359 (100)^b^		331 (100)		449 (100)				
	No	125 (65.4)		186 (51.8)		188 (56.8)		270 (60.1)				
	Yes	66 (34.6)		173 (48.2)		143 (43.2)		179 (39.9)				
**Free health screenings, n (%)**												.03
	Participants	191 (100)		359 (100)		331 (100)^b^		449 (100)^b^				
	No	82 (42.9)		137 (38.2)		156 (47.1)		167 (37.2)				
	Yes	109 (57.1)		222 (61.8)		175 (52.9)		282 (62.8)				
**Learn about stroke, n (%)**												<.001
	Participants	191 (100)^b,c^		359 (100)^d,e^		331 (100)^b,d^		449 (100)^c,e^				
	No	86 (45)		158 (44)		193 (58.3)		256 (57)				
	Yes	105 (55)		201 (56)		138 (41.7)		193 (43)				
**Learn about other health care topics, n (%)**												.002
	Participants	191 (100)		359 (100)^b^		331 (100)^b^		449 (100)				
	No	107 (56)		193 (53.8)		223 (67.4)		278 (61.9)				
	Yes	84 (44)		166 (46.2)		108 (32.6)		171 (38.1)				
**Speak to a health care provider, n (%)**												<.001
	Participants	191 (100)^b^		359 (100)^c^		331 (100)^b,c^		449 (100)				
	No	137 (71.7)		268 (74.7)		283 (85.5)		353 (78.6)				
	Yes	54 (28.3)		91 (25.3)		48 (14.5)		96 (21.4)				

^a^Except for the *P* value in the Age row, which is based on the Kruskal-Wallis test, all remaining *P* values are based on the chi-square test.

^b-e^Indicate significant differences in pairwise comparisons of either median scores or proportions in different ethnic groups. The presence of the same letter in the columns for two race or ethnic groups (ie, “a” in the Asian and “a” in the African-American race columns) indicate a significant difference between the median scores or proportions between those two respective ethnic groups.

During the 2018 and 2019 events, 2774 health screenings were completed within a period of 12 hours, averaging four health screenings per minute. These included blood pressure (1031/2774, 37.16%), stroke risk assessment (496/2774, 17.88%), bone density (426/2774, 15.35%), carotid ultrasound (380/2774, 13.69%), BMI (182/2774, 6.56%), serum lipids (157/2774, 5.65%), and hemoglobin A_1c_ (102/2774, 3.67%).

### Multimedia Engagement

Before the 2018 event, 27 Facebook posts were displayed between December 21, 2017, and April 23, 2018, reaching 17,975 people, with 782 likes, comments, and shares; 648 post clicks; 339 page likes; and 340 page followers. The hashtag *#stompoutstroke* achieved 51 posts and 951 likes, comments, and shares on Facebook; seven posts and 30 likes, comments, and shares on Twitter; and 36 posts and 2093 likes, comments, and shares on Instagram. The Stomp Out Stroke webpage [21] achieved 18,639 page views and 9316 unique visitors. A total of 20 media placements between March 25, 2018, and April 2, 2018, reached approximately 849,731 people in the Houston area. This included 12 television stories, five web-based news stories, two iQ radio interviews, and one print story (Multimedia Appendix 3).

## Discussion

### Principal Findings

This study provides novel insights regarding the implementation of Stomp Out Stroke, using multimedia engagement, followed by in-person stroke education and health screening initiative, among a large minority population disproportionately affected by large-scale flooding events and cerebrovascular disease. Stomp Out Stroke registrants were representative of Houston racial or ethnic demographics, including Hispanic or Latino (449/1401, 32.05%), African American (359/1401, 25.62%), and Asian (191/1401, 13.63%). Our population was young (median age 45 years), largely female, and had received more than a high school education. Overall, 16.99% (228/1342) of Stomp Out Stroke participants were uninsured, and 18.70% (251/1342) of the participants were insured through Medicare or Medicaid.

The low adoption rates of electronic consultations for cerebrovascular risk factors, such as hypertension and diabetes, leave questions about overall use and generalizability [32]. To our knowledge, a substantial number of completed health screenings has not been replicated in the literature; indeed, the frequency of four health screenings per minute speaks to the intervention fidelity and reproducibility of Stomp Out Stroke. The multimedia impact of Stomp Out Stroke is a critical component of our hybrid model; the use of social media, hashtags, our webpage, and strategic television, iQ radio, and web-based news stories reached nearly 850,000 people.

The American Heart Association has recently focused on the use of internet-based recovery strategies for stroke survivors [33]; however, there is a paucity of research on the impact of the health and well-being of the caregiver, who may share cerebrovascular risk factors. Overall, 20.79% (279/1342) of Stomp Out Stroke registrants were stroke survivors or caregivers, a population that is undersupported by the health care system and faces unique barriers to obtaining the needed services [34]. Disabled populations are disproportionately exposed to environmental health hazards and feel abandoned by the health care system due to the marginalization of services and a lack of knowledge or skills to reengage [35]. A 2019 study of stroke survivors and caregivers enrolled in an mHealth intervention showed that female caregivers were more likely to have unknown or poorly controlled cerebrovascular risk factors [36]. The combination of multimedia engagement, followed by in-person health screening, is a hybrid model that can be used in similar urban settings.

An analysis by the City of Houston estimated that 208,353 of 848,340 households were affected by Hurricane Harvey, with a disproportionate number consisting of racial or ethnic minorities and those of lower socioeconomic status [37]. At the request of the Federal Emergency Management Agency, the National Academies of Sciences, Engineering, and Medicine appointed a committee to hold workshops in Houston post Hurricane Harvey to gain an initial understanding of the impacts of urban flooding [38]. Residents most vulnerable to flooding were again described as minorities and the poor. Houston flood district funding comes from state and federal programs, focusing on capital improvement, structural projects, home buyouts, operations, maintenance, and repairs [39]. Unfortunately, the social aspects of urban flooding are far less studied than the physical aspects; indeed, rebuilding a bridge is tangible, and building health capacity is multifaceted. Data on the intangible impact of flooding on vulnerable populations, such as stroke screenings, provide another layer of urban flood risk assessments. Future data collection and analyses could help residents of flood-prone areas receive support from civic organizations, increasing their social agency and capacity.

Community engagement paradigms focused on stroke literacy help improve the awareness of signs and symptoms [40]. We found that Hispanic populations were more likely to attend free health screenings and that African American participants were more likely to express interest in learning about nonstroke health care topics compared with non-Hispanic White people. We also identified an increased interest in speaking to a health care provider among African American or Asian participants compared with non-Hispanic White participants. Differences in the provision of health care (ie, speaking to health care providers) do not necessarily reflect deficiencies. Our goal was to provide health assessments in multiple formats. By fulfilling specific health promotion and disease prevention needs, future community engagement interventions can be tailored to the needs of culturally diverse populations.

### Limitations

This study has some limitations. First, the organization and planning of Stomp Out Stroke is led by the Director of Stroke Community Outreach and Education, a full-time faculty member at the McGovern Medical School at UTHealth trained in Vascular Neurology. Her position is supported by endowment funds; sustaining Stomp Out Stroke requires collaborative efforts from multiple faculty members and neurology departmental support staff. Second, Stomp Out Stroke is funded through sponsorships, educational grants, philanthropy, and in-kind donations. Cost mitigation occurred by leveraging free educational resources at the local, state, and national levels. Third, a formalized emergency medical services and safety plan (Multimedia Appendix 4) was required for security, lost and found children, lost items, and medical emergencies. This plan was used in 2018 for a participant with transient ischemic attack symptoms. The proportion of female participants in Stomp Out Stroke (973/1390, 70%) was higher than the female population of Houston (1,162,454/2,320,268, 50.1%), which may increase type I error when addressing the health care needs of male participants. We used the largely accepted convention that ethnicity is self-identified, based on factors such as language and shared culture. Therefore, we did not analyze the differences between the Hispanic and non-Hispanic counterparts of each race. Statistical analyses of registrants detailed the purpose of attendance and health-related needs, with no linkage to cerebrovascular risk factors or health screening data. Finally, registrants were not asked about the extent of individual losses due to Hurricane Harvey. Future studies will include zip code mapping to identify households in flood-impacted areas.

### Conclusions

Stomp Out Stroke combined multimedia engagement with in-person health screenings to improve environmental justice for underserved populations at increased risk of urban flooding and cerebrovascular disease. The next step will focus on the use of mHealth technology to assess behavioral changes among repeat attendees, recurrent stroke among stroke survivors, and objective measures of stroke knowledge and preparedness.

## Appendix

Multimedia Appendix 1 Stomp Out Stroke program content.

Multimedia Appendix 2 Modified Framingham stroke risk profile—males and females.

Multimedia Appendix 3 Stomp Out Stroke multimedia report.

Multimedia Appendix 4 Stomp Out Stroke emergency medical services and safety plan.

## Abbreviations

mHealth: mobile health
UTHealth: University of Texas Health Science Center at Houston
