# Supplementary material for: Leveraging Multimedia Patient Engagement to Address Minority Cerebrovascular Health Needs: Prospective Observational Study
Source: J Med Internet Res. 2021 Aug 13;23(8):e28748. doi: 10.2196/28748 (PMC8398745; doi:10.2196/28748)
Supplement: Multimedia Appendix 1 [file jmir_v23i8e28748_app1.docx]

**
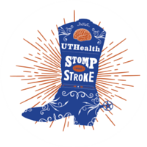
**

**2018 UTHealth**

**Stomp Out Stroke Festival**

**
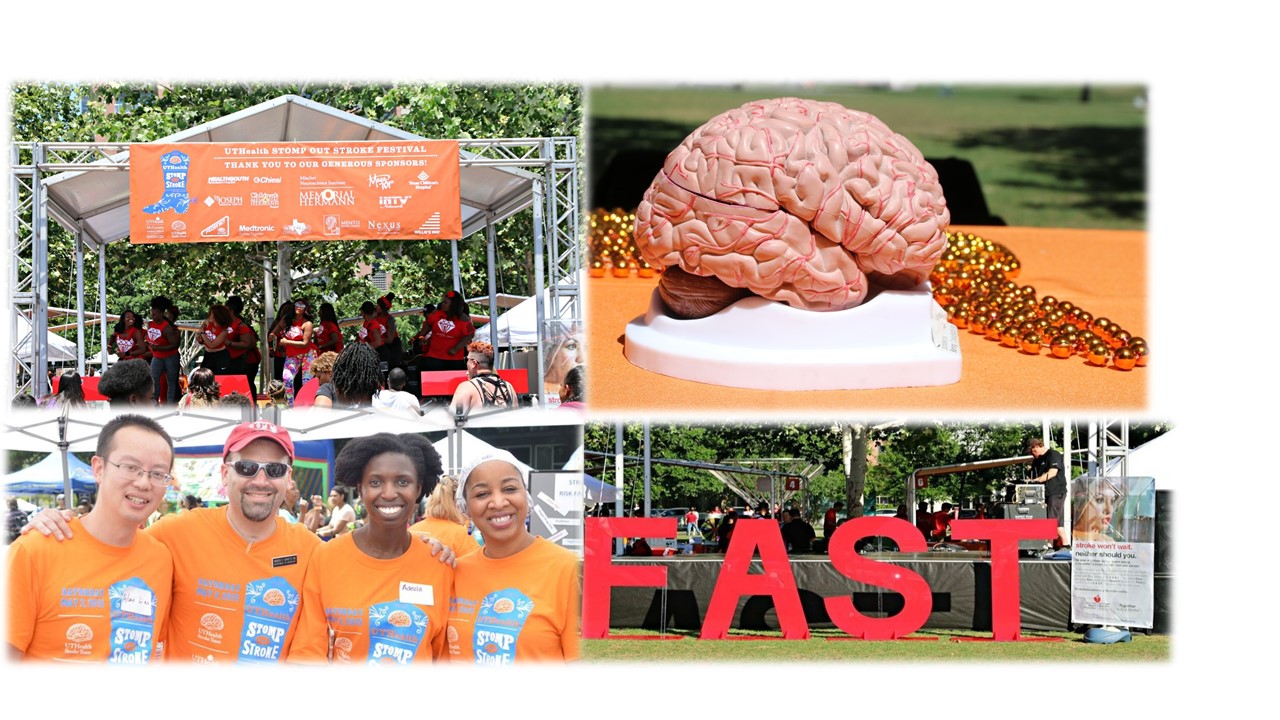
**


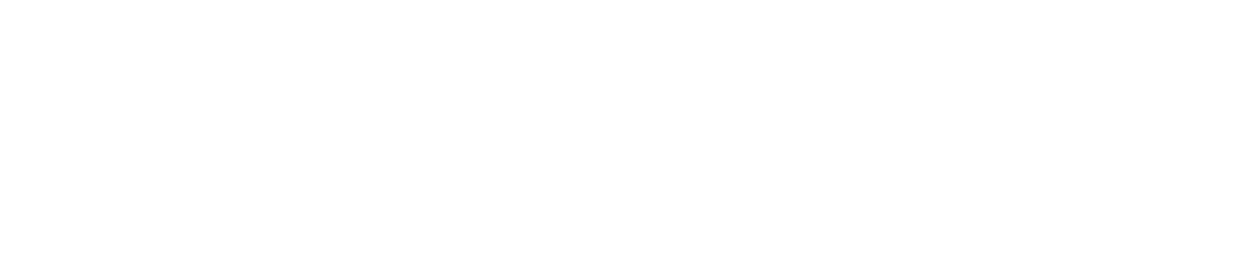


**Saturday, April 28, 2018**

**9:00 am - 3:30 pm**

**Jones Lawn, Discovery Green**

**1500 McKinney St. 77010**

**
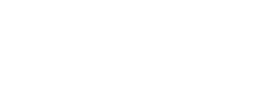
**

**CENTRAL ZONE**

**1. Guest Check-in** For pre- registered guests. Opens @ 8am.

**2. Girl Scouts Check-in**

All Girl Scout troops check-in here from 8am -9am.

**3. AmeriBrain Exhibit UTHealth** Inflatable brain. See the brain stem, cerebellum and areas of the brain for hearing, touch movement and personality.

**4. Girl Scouts: Hula Hoop Fitness UTHealth** Girl scouts have fitness fun with hula hooping. 8:30am-12:30pm

**5. Girl Scouts: Stomp Out Stroke** **UTHealth** Girl

Scouts learn about stroke & stroke signs using FAST. 8:30am -12:30pm

**5. Miss Moo** Houston Livestock Show and Rodeo™ 11:30am- 2:30pm.

**6. Stroke Risk Factors Baylor St. Luke’s Medical Center** Learn about high blood pressure, high cholesterol and diabetes and how each relates to stroke.

**7. Concussions NAHN Houston Chapter/ UTHealth.** Learn the signs and treatment for concussions.

**8. Brain Parts & Cranial Nerves UTHealth Neurology Residents** Learn about the parts and function of the brain. See brain models.

**9. Willie’s Way** Learn about our foundation dedicated to improving the lives of stroke survivors through education, support and fundraising. Meet Willie, a 10 year stroke survivor with an amazing success story and founder of Willie’s Way.

**10. University of Houston Brain-Computer Interface Systems** Dedicated to engineering of the brain, design of non-invasive brain-machine interface and robotic systems for repair of the motor system. See robotic demos.

**11. Onsite Registration** For walk in guests. Opens @ 8am.

**12. Brain Imaging. Dept of Diagnostic & Interventional Imaging UTHealth** Imaging to see the brain structure and function: CT, MRI and fMRI.

**13.** **Nutrition and the** **Brain Memorial Hermann Southwest Clinical Nutrition** Healthy eating, learn the food groups. See a Sugar Shocker Display.

**14. Medical Fitness Pros**

Providing medical exercise for those with neurological conditions.

**15. Healthy Brain Aging UTHealth Geriatrics** Steps to promote a healthy brain as you age.

**16. Cooking Healthy with Chef Tim Sadler. American Heart Association** Demo with samples @ 11:30 am & 1:30 pm.

**17. My Life Check Life's Simple 7 American Heart Association** Assess your current cardiovascular health.

**18. Guest Check- In**

Check-in for pre-registered guests.

**19. Encompass Health Rehabilitation Technology** One of the nation's largest rehabilitation providers. Onsite therapists will demonstrate rehabilitation technologies such as Bioness.

**20. The Health Museum** Presenting an interactive presentation with a hands-on sheep brain dissection @ 12:40 pm and 1:15 pm.

**21. Volunteer & Exhibitor Check-in.**

**22. Mobile Stroke Unit Frazer Ltd.** See one of the first emergency vehicles designed to treat stroke patients in the US!

**_____________________**

**STAGE ZONE**

**Guest Speaker, José Griñan**

**Co-Emcee, Lady MaCallan**

**23. Medical and Cooling Station Southeast Texas Regional Advisory Council (SETRAC)** Onsite BLS level emergency medical support or drop by to cool down.

**24. Stage Station**

Media and Performers

Check-in.

**25. DJ PANCAKE** Music provided by Houston's own DJ PANCAKE with Pinto

Productions Entertainment.

**26. Door Prizes & Brain Game: Lollipop Tree.** Door

prizes drawing every 30 minutes. Must be present to win.

**27. Popcorn & Snacks** Free snacks while supplies last.

**28. Water Station**

**Ariana The Realtor**

Free bottled water, while supplies last.

**29. MPOWER Expert Fitness & Nutrition**

Results driven fitness and nutrition solutions with a focus on body fat reduction.

**30. Tobacco Prevention MD Anderson Cancer Center** Tobacco prevention and cessation.

**_____________________**

**HEALTHY BRAIN ZONE**

**31. Kidney Health Screen**

**Texas Kidney Foundation.** Check Blood pressure, glucose and eGFR (kidney function).

**32. Blood Pressure (BP) Screen. UT Physicians** BP check. Review results with a health care provider.

**33. Bone Density Screen**

**UT Physicians** Back pain, stooped posture or fracture history, maybe signs of osteoporosis. Check your heel bone density.

**34. Carotid Ultrasound UTHealth Stroke Team** Assesses blood flow and detects blockages in the carotid arteries. For adults aged >55 years + one stroke risk factor.

**35. Carotid Ultrasound UTHealth CV Surgery** Assesses blood flow and detects blockages in the carotid arteries. For adults aged >55 years + one stroke risk factor.

**36. Cholesterol, Glucose and Blood Pressure Screenings HEB Pharmacy** Check your cholesterol- no fasting required. Glucose- fasting required for accuracy. Review results & Q&A with pharmacists.

**37. Ask the Pharmacist**

**MH TMC Pharmacy Services/ VAPhA**

Ask drug related questions, review medications, ask about generic drugs or pharmacy services.

**38. Optical Illusions UTHealth Medical Students** See some optical illusions and discover how the brain can interpret images from the eyes.

**39. Fitness Testing- HANP**

How much should you exercise daily? + Fitness testing.

**40. Ask the Dietician! Memorial Hermann-TMC Nutrition Dept.** Tips for healthy eating. Check BMI and body fat percentage.

**41. Stroke Risk Assessments. UTHealth Stroke Team** Learn about stroke prevention, treatment and signs of stroke. Check your stroke risk. **_____________________**

**STROKE RECOVERY ZONE**

**42 Mentis Neuro Health** Providing the highest

quality post-acute neuro-rehabilitation to persons who have sustained an acquired brain injury.

**43. Fall Risk**

**Mentis Neuro Health**

Fall prevention, fall risk, reduce fall risk. Take the Timed Get up and Go Test.

**44. Mega 101**

Meet our team who will be passing out goodies from

9am – 12pm.

**45. DLG Ice Factory** Specializing in the art of Ice sculpting. Drop by to see what we are sculpting.

**46. Obstacle Course UTHealth** Must be > 5 years old & a parent present.

**47. Texas Snakes**

Meet several species of nonvenomous snakes. Guests may touch, hold or take a pic with safe, friendly snakes

9:00 am -12:00 pm.

**48. Memory Screen & Forgetfulness**

**UTHealth Neurology**

Memory loss? Forgetful? Memory screen for adults, age > 45.

**49.** **Atrial Fibrillation (AF) UTHealth** What is AF? How does AF relate to stroke? Afib treatment.

**50. National Stroke Association** Providing stroke education and programs to stroke survivors, caregivers, and healthcare professionals.

**51. Obstructive Sleep Apnea (OSA)**

**St. Joseph Medical Center** What is OSA? OSA and stroke risk. Take the Stop Bang screen.

**52. Home Exercise Programs. PAM Rehabilitation Hospital of Clear Lake.** 45 bed inpatient hospital specializing in neurorehabilitation.

**53. Texas Heart Institute’s Center for Women’s Heart & Vascular Health** Everything we do to reduce heart attack risk, reduces stroke risk—yet women don’t perceive themselves at risk for stroke. Prompt diagnosis and treatment maybe the difference between life and death. Drop by at 11:10 am & 2:00 pm for “Do You Know Your Risk?” with Dr. Stephanie Coulter & Dr. Karla Campos (En Española). Screenings 9am-11am only

**54. Houston Aphasia Recovery Center**

Wellness program and support services for persons with aphasia and their families.

**55. Yoga Sessions. UTHealth** Receive information and participate in a demo. Chair Yoga- 9:30am, 10:30am, 11:30am, 12:30pm for stroke survivors, seniors, brain injured. Yoga- 10am, 11am, 12pm, 1pm for general public. Must be age >12 years.

**56. Diabetes Awareness and Wellness Network (DAWN)** Diabetes risk assessments and health coaching.

**57. Stroke Warriors**Local stroke support group, welcoming new members.

**58. Encompass Health**

One of the nation's largest rehabilitation providers. Get info on rehabilitation services, programs and technology.

**59. Communication Sciences & Disorders- University of Houston**

Do you have a language or hearing problem? Learn

about our research &

clinical services.

**60. Hula-Hoop Fitness**

**UThealth** Exercise for the entire family. Free hula hoops while supplies last.

**_____________________**

**CHILDREN’S ZONE**

**61. Texas Heart Institute’s Project Heart** Learn about heart and circulatory system anatomy. Why making healthy eating choices and physical activity is important to the circulation system. Earn a Cool-E badge.

**62. Kids Think FAST Houston Methodist Neurological Institute** Kids learn about stroke and signs of stroke using FAST. 9am -12:30pm

**63. Pediatric Stroke & Seizures** Causes of stroke in children? What is a seizure? Why kids have seizures?

**64 Five Senses and the Brain UTHealth** Interactive activities to teach kids about the five senses.

**65. Bubble Fun**

**UTHealth** Drop by to blow bubbles. Free bubble bottle while supplies last.

**65. Balloon Twister UTHealth** Free balloon art. 10am-3pm.

**66. Bike and Helmet Safety Center for Childhood Injury Prevention Texas Children’s Hospital** Learn Bike & helmet safety for kids.

**67. Texas Medical Association's Hard Hats for Little Heads Michael E. DeBakey Veterans Affairs Medical Center in Houston** Properly fitting helmets can prevent head injuries. Learn helmet safety and fitting. Free bike helmet while supplies last.

**68. Parachute Fun UTHealth**

Fun and fitness with a

giant parachute.

**69 Kids Characters UTHealth**

Meet & take pics with a favorite kid's cartoon character.

**70. Kids Vision Screen**

**Eye Care For Kids**

Check your child's vision. Testing visual acuity and general eye health examination.

**71. Face Painting**

**Tracy's Magic Mirror**

Free face painting with fun, cool designs!

**72. Brain on Drugs**

**Center forNeurobehavioral Research on Addiction UTHealth** Learn the effects of different drugs on the brain. Spin a “Quiz Wheel” on drug awareness & win a prize.

**73. Kids Migraines THINK- The Houston Institute of Neurology for Kids**

Does your child complain of frequent headaches? Diagnosis and treatment of migraines in kids.

**73. Balloon Twister**

**THINK Neurology**

Drop by and get a free snow cone and kids meet our balloon twister

**_____________________**

**LAMAR STREET**

**74. St. Joseph Medical Center Angioscreen Mobile Health Unit** Carotid Doppler, abdominal aortic aneurysm and ankle brachial index screenings.

**75. CHRISTUS Healthy Living Mobile Clinic.**

Blood pressure, glucose and cholesterol screenings and health education.

**Meet Our Emcees**

**José Griñan**

Anchors FOX 26 Morning and Midday Newscasts for the past 20+ years. He previously hosted "Hola Houston" and continues to host "The Black Voice", two weekly public affairs programs. Jose stays active in the community, volunteering his time for a number of non-profit and cultural, educational and social service organizations. His career choice has allowed him to be an eyewitness to both triumph and tragedy in today’s world. Jose is married to Kathryn Griffin, and they are the proud parents of five daughters.

**Lady MaCallan**

Houston’s own red headed powerhouse. She is a model, emcee and community advocate, with over 15 years of experience serving as a volunteer and board member of local nonprofit organization Lady is a proud mother and recently founded the Fancy Hat Society, a networking and empowerment group for women.

**STAGE SCHEDULE**

8:30 am DJ PANCAKE

10:00 am Lion Dance Team

10:45 am Ready Heroes

11:05 am Welcome Remarks:

Dr. Noser & José Griñan

11:30 am McTeggart Irish Dancers of South Texas

12:10 pm Crystal Wall’s MixFitz

TurnUp Fitness

12:50 pm Young Lyric, Houston's

"Hip Hop Princess **“**

1:15 pm Soundbox Studios

1:30 pm Shingari's School of Rhythm

2:00 pm Too Cool to Smoke Puppet Show

2:15 pm Faces of Stroke:

Ellie Cammack & Willie Leftwich

2:45 pm Mixteco Ballet Folklorico

3:15 pm DJ PANCAKE

[
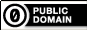
](https://urldefense.proofpoint.com/v2/url?u=https-3A__creativecommons.org_publicdomain_zero_1.0_&d=DwMGaQ&c=bKRySV-ouEg_AT-w2QWsTdd9X__KYh9Eq2fdmQDVZgw&r=ybtSbaUF5ARWjsRA1Py33gG9h4Fgij_mqU3t_y0s3g0&m=uRpq2sGMqNh8ykqF0doNTfkmhMPmsq0Ubv7NMcG5Qos&s=04gi-YPuyvVLNDPuej7R3NPDyl9omVQi4wjyW105Z4M&e=)

_To the extent possible under law,_[_Elizabeth Noser_](https://urldefense.proofpoint.com/v2/url?u=https-3A__www.strokefestival.org_uploads_1_1_3_1_11311015_final-5F2019-5Fprogram-5Fnoser.pdf&d=DwMGaQ&c=bKRySV-ouEg_AT-w2QWsTdd9X__KYh9Eq2fdmQDVZgw&r=ybtSbaUF5ARWjsRA1Py33gG9h4Fgij_mqU3t_y0s3g0&m=uRpq2sGMqNh8ykqF0doNTfkmhMPmsq0Ubv7NMcG5Qos&s=SuDBf5obYeik6IXU_Z6GKmQnDoKuGP5YJDiup2RoPQ8&e=)_has waived all copyright and related or neighboring rights to Stomp out Stroke Festival Program - 2018. This work is published from: United States._
