# Supplementary material for: Leveraging Multimedia Patient Engagement to Address Minority Cerebrovascular Health Needs: Prospective Observational Study
Source: J Med Internet Res. 2021 Aug 13;23(8):e28748. doi: 10.2196/28748 (PMC8398745; doi:10.2196/28748)
Supplement: Multimedia Appendix 2 [file jmir_v23i8e28748_app2.pdf]

UTHealth Stroke Team

University of Texas Medical School at Houston

Stroke Risk Assessment Tool / Formulario de evaluación de Riesgo Cerebrovascular

Name/Nombre:__________________Age/Edad:____ Hypertension/Hipertensión (yes/si)(no)______On medication (yes/si)(no)

Modified Framingham Stroke Risk Profile

Men/Hombres:

Age/Edad:
0(54-56) +1(57-59) +2(60-62) +3(63-65) +4(66-68) +5(69-72) +6(73-75) +7(76-78) +8(79-81) +9(82-84) +10(>=85)

Systolic BP, untreated/Presión sistólica, no tratada (mmHg):
+1(106-115) +2(116-125) +3(126-135) +4(136-145) +5(146-155) +6(156-165) +7(166-175) +8(176-185) +9(186-195) +10(196-205)

Systolic BP, treated/Presión sistólica, tratada (mmHg):
+1(106-112) +2(113-117) +3(118-123) +4(124-129) +5(130-135) +6(136-142) +7(143-150) +8(151-161) +9(162-176) +10(177-205)

History of Diabetes/Historia de Diabetes:
0 = No +2 = Yes/Si

Smoking/Tabaquismo:
0 = No +3 = Yes/Si

Cardiovascular disease/Enfermedad cardiovascular:
0 = No +4 = Yes/Si

Atrial fibrillation /Fibrilación atrial:
0 = No +4 = Yes/Si

Metabolic Sindrome ATP III criteria

Three or more of the following/Tres o más de los siguientes:
1. Abdominal obesity (waist circumference)/Obesidad Abdominal (circumferencia de la cintura):______
>102 cm or >40 in
2.Triglycerides/Triglicéridos:_____
>=150mg/dL
3. HDL Cholesterol/Colésterol HDL:_____
<40mg/dL
4. Blood pressure/Presión arterial:_____
>=130/>=85 mmHg
5. Fasting glucose/Glicemia en ayunas:_____
>= 100mg/dL

Recomendations/Recomendaciones:

0-4pts: Low/bajo; 5-10pts: Moderate/moderado; >10pts: High/alto

Participant Number_____

Adapted from: Goldstein LB, et al. Primary Prevention of Ischemic Stroke. Circulation 2006;113;873-923

UTHealth Stroke Team

University of Texas Health Sciences Center at Houston

Stroke Risk Assessment Tool / Formulario de evaluación de Riesgo Cerebrovascular

Name/Nombre:___________________Age/Edad:____Hypertension/Hipertensión (yes/si)(no)______On medication (yes/si)(no)

<u>Modified Framingham Stroke Risk Profile</u>

Women/Mujeres:

Age/Edad:
0(54-56) +1(57-59) +2(60-62) +3(63-64) +4(65-67) +5(68-70) +6(71-73) +7(74-76) +8(77-78) +9(79-81) +10(82-84)

Systolic BP, untreated/Presión sistólica, no tratada (mmHg):
+1(95-106) +2(107-118) +3(119-130) +4(131-143) +5(144-155) +6(156-167) +7(168-180) +8(181-192) +9(193-204) +10(205-216)

Systolic BP, treated/Presión sistólica, tratada (mmHg):
+1(95-106) +2(107-113) +3(114-119) +4(120-125) +5(126-131) +6(132-139) +7(140-148) +8(149-160) +9(161-204) +10(205-216)

History of Diabetes/Historia de Diabetes:
0 = No +3 = Yes/Si

Smoking/Tabaquismo:
0 = No +3 = Yes/Si

Cardiovascular disease/Enfermedad cardiovascular:
0 = No +2 = Yes/Si

Atrial fibrillation /Fibrilación atrial:
0 = No +6 = Yes/Si

<u>Total ______</u>

<u>Recomendations/Recomendaciones:</u>
0-4pts: Low/bajo; 5-10pts: Moderate/moderado; >10pts: High/alto

Decreasing your risk

1.
2.
3.

Adapted from: Goldstein LB, et al. Primary Prevention of Ischemic Stroke. Circulation 2006;113;873-923
