# Supplementary material for: Leveraging Multimedia Patient Engagement to Address Minority Cerebrovascular Health Needs: Prospective Observational Study
Source: J Med Internet Res. 2021 Aug 13;23(8):e28748. doi: 10.2196/28748 (PMC8398745; doi:10.2196/28748)
Supplement: Multimedia Appendix 3 [file jmir_v23i8e28748_app3.pdf]

Stomp Out Stroke Festival Multimedia Report

Social Media/ Website
Facebook page:

- 27 posts between December 21, 2017 - April 23, 2018
- 17,975 People reached
- 782 Likes, comments and shares
- 648 Post clicks, 339 page likes, 340 page followers

#stompoutstroke
Facebook: 51 posts, 951 likes, comments and shares
Twitter: 7 posts, 30 likes, comments and shares
Instagram: 36 posts, 2093 likes, comments and shares

Stroke Festival website activity: www.strokefestival.org
18,639 Page views, 9,316 Unique visitors

Total 20 media placements between March 25 and April 28 includes 12 television stories, 5 online stories, 2 radio interviews and one print story.

Online News: 5
Publications (LexisNexis): 1
Television: 12
iQ Radio: 2

Total Audience: 849,731
Media Value: $24,251.45

Online News (5, Audience: 439,442, $264.26)

APR 28 **UTHealth Stomp Out Stroke Festival**
3:38 PM CST
tmc.edu
Join the UTHealth Stroke Team at the 6th Annual STOMP OUT STROKE FESTIVAL Saturday, April 28, 9 am-3:30 pm at Discovery Green, 1500 McKinney St.

APR 28 **UTHealth Stomp Out Stroke Festival**
5:06 AM CST
culturemap.com
Photo courtesy of The University of Texas Health Science Center at Houston (UTHealth) Participants of the will learn lifesaving information about stroke and brain health, enjoy free health screenings, fitness & nutrition demonstrations and Q&A with stroke physicians, nurses and pharmacists. This

Aud:11,128 $6.95

APR 26 **Family Fun: Cars, crawfish and Comicon**
10:03 AM CST
chron.com
Image 1 of 8 Dance of Asian America will perform East Meets West XVI at Miller Outdoor Theatre on Saturday. Dance of Asian America will perform East Meets West XVI at Miller Outdoor Theatre on Saturday. Photo: Li Jia / Li Jia Image 2 of 8 Sample over 72 craft beers from 24 breweries and

Aud: 345,206 $215.75

APR 23 **Gerson Suarez-Cedeno, M.D., on KXLN Houston Channel 45**

8:24 AM
CST

univision.com

Dr. Gerson Suarez-Cendeno promoted the Stomp Out Stroke Festival.

Aud: 41,554 $20.78

APR 9
5:22 PM
CST

Gerson Suarez-Cedeno, M.D., on Univision Houston Channel 45

univision.com

Gerson Suarez-Cedeno, M.D., on KXLN Channel 45 to promote UTHealth Stomp Out Stoke Festival.

Aud: 41,554 $20.78

Publications (LexisNexis) (1)

APR 28
2:11 AM
CST

FAMILY FUN

The Houston Chronicle

Comicon Visit with comic book, sci-fi and cosplay dealers along with artists from all over the world at Trader's Village. When: 10 a.m.-5 p.m. Friday and Saturday Where: 7979 N. Eldridge Details: $4 parking fee; tradersvillage.com East Meets West XVI Traditional and contemporary Asian dance performances,

TV (12, Aud: 410,289, $23,987.19)

APR 28
10:00 PM
CST

FOX 26 News at 9

Houston

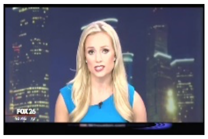

WELL, TODAY IN DOWNTOWN HOUSTON IT WAS A SIXTH ANNUAL STOMP OUT STROKE FESTIVAL. ORGANIZERS SAY TODAY WAS ALL ABOUT STROKE AWARENESS AND ROCKIESING THE SYMPTOMS OF A STROKE. FOX 26 MORNING NEWS ANCHOR JOSE GRENOGN WAS THERE AND TOLD

Aud: 48,137 $2,949.17

APR 28
5:00 PM
CST

Gerson Suarez-Cedeno, M.D., on Telemundo Houston Channel 47

Houston

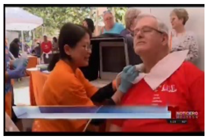

♪♪ ♪♪♪♪ ♪♪(MUSICA)♪♪ >>> HOY SE REALIZO UN GRAN FESTIVAL EN (NOMBRE EN INGLES) PARA CONCIENTIZAR A LA COMUNIDAD SOBRE LOS DERRAMES CEREBRALES, INICIO A LAS NUEVE DE LA MA}ANA Y TERMINO PASADAS LAS TRES DE LA TARDE, SE OFRECIERON EVALUACIONES

Aud: 17,808 $564.58

APR 28
8:00 AM
CST

Today

Houston

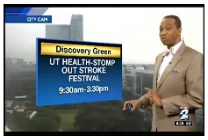

BUT THIS MORNING, AT DISCOVERY GREEN, U.T. HEALTH IS SPONSORING THE STOMP OUT STROKE FESTIVAL. GO OUT THERE. YOU GET FREE HEALTHNGS AND INFORMATION YOU WHAT -- INFORMATION ON WHAT YOU NEED TO KNOW. WE GOT THAT. WE GOT THE BP MS 150 GOING ON AS WELL. HERE'S THE SHOT OUT AT

Aud: 78,339 $2,692.95

APR 28 FOX 26 Morning News Weekends 7am

7:00 AM
CST

Houston

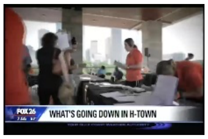

IT WILL BE SATURDAY FROM 10:00 A.M. UNTIL 11:00 P.M. SO STOP BY ANY TIME WITH THE FAMILY AND IT'S FREE TO ATTEND. KEEP THE HEALTHY THEME GOING THIS SATURDAY AT THE STOMP OUT STROKE FESTIVAL. TAKE THE WHOLE FAMILY TO LEARN ABOUT SIGNS OF STROKES, BRAIN ANATOMY AND MORE. IT'S FROM 9:00

Aud: 25,978 $893.01

APR 28 FOX 26 Morning News Weekends 5am

5:00 AM
CST

Houston

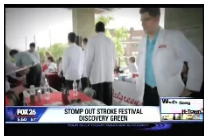

SO STOP BY ANY TIME WITH THE FAMILY AND IT'S FREE TO ATTEND. KEEP THE HEALTHY THEME GOING THIS SATURDAY AT THE STOMP OUT STROKE FESTIVAL. TAKE THE WHOLE FAMILY TO LEARN ABOUT SIGNS OF STROKES, BRAIN A ANATOMY AND MORE. IT'S FROM 9:00 UNTIL 3:30 AND FREE TO A TEND. THIS SUNDAY, A COOK

Aud: 6,829 $234.78

APR 25 FOX 26 News at 8AM

8:00 AM
CST

Houston

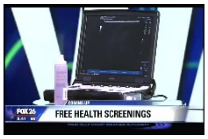

WEEKEND. I'M ABOUT TO UNDERGO THREE OF THEM. ONE INCLUDES AN ULTRASOUND OF THE NECK. SEE IT LIVE ON AIR AHEAD OF THE STOMP OUT STROKE FESTIVAL JUST AHEAD. BUT FIRST The savings are in full bloom at Ross. If you're looking for an incredible selection of the brands you love, this season's

Aud: 52,649 $997.33

APR 25 FOX 26 News at 7AM

7:00 AM
CST

Houston

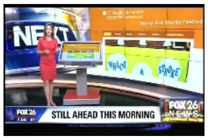

COMING UP AT 8:00, A NEW HEART DISEASE SCREENING IS ONE OF SEVERAL FREE TESTS THAT YOU CAN TAKE ADVANTAGE OF GETTING AT THIS WEEKEND'S STOMP OUT STROKE FESTIVAL. YOU'LL HAVE A DOCTOR HERE LIVE TO DEMONSTRATE SOME OF THE TESTS. IT'S ALL COMING UP IN THE 8:00 HOUR. WEL SEE YOU THEN.

Aud: 50,830 $962.89

APR 23 FOX 26 News at 8AM

8:00 AM
CST

Houston

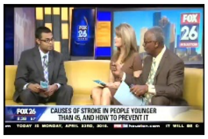

UNDERLYING CAUSES SUCH AS CLOSING THE PFO. >> Jose: THIS WEEKEND THERE WILL BE STOMP OUT STROKE. I'LL BE THERE. TELL US ABOUT THE FESTIVAL. >> SO U.T. IS ORGANIZING THIS EVENT. IT'S CALLED THE STROKE FESTIVAL OR STOMP OUT STROKE FESTIVAL. IT'S BEING ORGANIZED ON SATURDAY SATURDAY,

Aud: 54,470 $1,031.84

APR 23 Gerson Suarez-Cedeno, M.D., on Univision Houston Channel 45

5:00 AM
CST

Houston

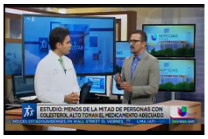

ACUMULA EN CANTIDADES ANORMALES. -- QUE SE LIBERA EN EL CEREBRO Y QUE SE ACUMULAN CANTIDADES ANORMALES. UN PORCENTAJE ELEVADO DE PERSONAS QUE A TOMAR LOS

MEDICAMENTOS -- UN PORCENTAJE ELEVADO DE PERSONAS NO TOMAN LOS MEDICAMENTOS ADECUADOS PARA EL COLESTEROL. ESTAMOS CON UN DOCTOR QUE NOS HABLAR'acute;

Aud: 9,329 $1,441.05

APR 21 Lauren Fournier, M.D., on FOX 26 News

7:00 AM CST

Houston

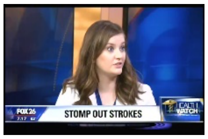

Leading causes of death and disability in seniors across the United States. that'S because the likelihood of having a stroke nearly doubles every 10 years after the age of 55. Because FOX 26 is your station for help, we have Dr. Lauren here from Hermann Texas Medical Center to join us. Good

Aud: 21,348 $4,647.70

APR 14 Gerson Suarez-Cedeno, M.D., on Univision Houston Channel 45

5:00 PM CST

Houston

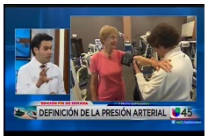

PRESIDENTE DONALD TRUMP DECLAR_ ESTAR S'acute;BADO EL ATAQUE CONTRA SIRIA COMO UNA "MISI_N CUMPLIDA". Y TODOS LOS D^ AS EN ESTADOS UNIDOS LA PRESI_N ARTERIAL ALTA COBRA LA VIDA DE APROXIMADAMENTE 1000 PERSONAS INCLUYENDO UN ATAQUE CEREBRAL, CARDIACO O INSUFICIENCIA CARDIACA O

Aud: 35,243 $6,824.73

APR 9 Gerson Suarez-Cedeno, M.D., on Univision Houston Channel 45

5:00 AM CST

Houston

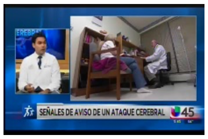

NI}OS COMO ADULTOS. M_NICA NAVARO, UNIVISI_N. RODOLFO: EL DERRAME CEREBRAL ES LA QUINTA CAUSA DE MUERTE Y UNO DE LOS PRINCIPALES MOTIVOS DE DISCAPACIDAD EN ESTADOS UNIDOS Y ES LA CUARTA CAUSA DE MUERTE EN EL ESTADO DE TEXAS. EST'acute; CON NOSOTROS EL DOCTOR HERSON SUARES CEDE}O. VAMOS

Aud: 9,329 $747.16

iQ Radio (2)

APR 28 KTRHAM 740 Houston - Gene Green

12:00 PM CST

Houston

KTRHAM - to be aware of we have an error quality of learnt in effect for today kelso have high grass pollen be aware that if you'd be out doing some gardening is seventy seven really warming up in dallas do now is that what was it you have got eighty that carries it seventy nine now in katie in it's eighty at

Market Rank: 10

MAR 25 Lauren Fournier, M.D., on KPRC Radio's Fifty = Show

4:00 PM CST

Houston

KTRHAM - next up a discussion of whirled leading cause of death and disability here in around the world especially among senior likelihood of stroke is all this statistic in my preparatory work in it called mean little on work like that of stroke merely doubles every ten years after fifty by the pure seventy

Market Rank: 10
