# Supplementary material for: Leveraging Multimedia Patient Engagement to Address Minority Cerebrovascular Health Needs: Prospective Observational Study
Source: J Med Internet Res. 2021 Aug 13;23(8):e28748. doi: 10.2196/28748 (PMC8398745; doi:10.2196/28748)
Supplement: Multimedia Appendix 4 [file jmir_v23i8e28748_app4.docx]

# UTHEALTH STOMP OUT STROKE FESTIVAL MEDICAL EMERGENCY, SECURITY & SAFETY PROTOCOLS

During any large community event, we need to be prepared for unexpected situations that may arise. We want to keep safety in mind at all times and be aware of certain procedures in place for the safety of our volunteers, exhibitors, event organizers and our community guests. Please review and make note of the below procedures that all volunteers and exhibitors should be aware of.

# ESTABLISH AVAILABLE MEDICAL ASSETS

**9-1-1 Access**- Event organizers, medical station and police officers will have the capability on- site to directly notify 9-1-1 via telephone in the event of any medical emergency.

**Basic Life Support (BLS) Medical Aid Station with EMTs:** A tent will be in a fixed location at the venue entry, where medical staff has the ability to provide BLS level care staffed by at least two

(2) certified Emergency Medical Technicians and 1 physician. Examples of BLS care are cleaning, bandaging and localized simple wounds such as scrapes and shallow cuts, providing cold packs for musculoskeletal strains and bruises, and giving drinking water and a place to rest for patients who are mildly dehydrated.

Will have 9-1-1 communications capability via cell phone, sign marked Medical Aid Station which will be on site map and designated Medical & Safety Lead Physician who will oversee all medical care delivered by program personnel.

**Medical Bike Teams:** Mobile medical teams consist of two (2) or more personnel, one of whom must be an EMT or higher level provider (i.e. Paramedic, Registered Nurse, etc) with treatment supplies necessary for the provider’s skill level & event type, and communications capability with the Medical Aid Station at the event.

**MEDICAL EMERGENCY**: In the event of any medical emergency, you (or a volunteer if readily available) should stay with the guest and send a team member from your station or a volunteer to immediately notify your Zone Coordinator of the medical emergency. The Zone Coordinator will radio the onsite medical station, and the medical team will come to you to evaluate the guest. If this is a serious medical emergency, the medical station team will notify our onsite EMS personnel who can provide BLS and can safely transport the guest via ambulance to the nearest hospital.

pg. 1

**SECURITY**: We will have three off duty Houston Police Department officers on site at all times (7AM-4PM). One officer will be guiding traffic on Las Avenidas, one stationed at the entry of the event in the Central Zone and one stationed on Jones Lawn. In the event of any potential or ongoing security issues, you should notify your Zone Coordinator. The Zone Coordinator will immediately radio on site security of the issue and need for security assistance.

**LOST CHILD**: At event registration, all guests are given an orange wristband to wear. For all minors under the age of 16, the parent’s last name and cell phone number will be written on their band. If a lost child approaches you, remain calm. You (or volunteer if readily available) should stay with the child and another team member from your station or volunteer should immediately notify the Zone Coordinator. The Zone Coordinator will put the child's name and description out over the radio. The Zone Coordinator will then call the parent’s number listed on the wristband. If no answer or the wristband is not in place, we will ask the minor for their parents name and make an announcement on the PA system calling for that individual.

**MISSING CHILD REPORTED**: if a parent approaches you, reporting that their child is missing remain calm and immediately report this to your Zone Coordinator. The Zone coordinator will need to know the child's name, age, description and where the child was last seen. The Zone Coordinator will then radio a message that a child is missing and include the child's name, age, description and where the child was last seen. All other radio operators are to move towards the last known location the child was seen while other volunteers will be directed to watch the perimeter. If needed, the alert will be broadcast from the Festival stage. Once found, the Zone Coordinator is to radio that the child was found and the child is to be brought to the Medical Station. The original volunteer who was approached by the parent is to escort the parent to the Medical station so that they can be re-united with the minor as quickly as possible.

**Extreme Weather Conditions Plan:** This is a rain or shine event. Contingency plan should an extreme weather event occur. During a hurricane, event will be canceled. During a tornado watch or warning, your health and safety lead will direct participants to points of shelter. Will have designated person to monitor local weather information for the latest updates during the event.

pg. 2

[
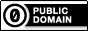
](https://creativecommons.org/publicdomain/zero/1.0/)
To the extent possible under law, Elizabeth Noser has waived all copyright and related or neighboring rights to this document: Emergency and Security Protocol.

We [Elizabeth A Noser and Nneka L Ifejika], The First and Corresponding Authors of this article contained within the original manuscript which includes any diagrams & photographs and any related or stand alone film submitted (the Contribution”) has the right to grant on behalf of all authors and does grant on behalf of all authors, a licence to the “BMJ Publishing Group Ltd” (“BMJ”) and its licensees, to permit this Contribution (if accepted) to be published in any BMJ products and to exploit all subsidiary rights, as set out in our licence set out at:

<http://group.bmj.com/products/journals/instructions-for-> authors/wholly_owned_licence.pdf

pg. 3
